# Mutations and Modeling of the Chromatin Remodeler *CHD8* Define an Emerging Autism Etiology

**DOI:** 10.3389/fnins.2015.00477

**Published:** 2015-12-17

**Authors:** Rebecca A. Barnard, Matthew B. Pomaville, Brian J. O'Roak

**Affiliations:** ^1^Department of Molecular & Medical Genetics, Oregon Health & Science UniversityPortland, OR, USA; ^2^Department of Biology, California State UniversityFresno, CA, USA

**Keywords:** autism, autism spectrum disorder (ASD), CHD8, systems biology, co-expression networks, functional genomics, subtype, *de novo* mutations

## Abstract

Autism Spectrum Disorder (ASD) is a common neurodevelopmental disorder with a strong but complex genetic component. Recent family based exome-sequencing strategies have identified recurrent *de novo* mutations at specific genes, providing strong evidence for ASD risk, but also highlighting the extreme genetic heterogeneity of the disorder. However, disruptions in these genes converge on key molecular pathways early in development. In particular, functional enrichment analyses have found that there is a bias toward genes involved in transcriptional regulation, such as chromatin modifiers. Here we review recent genetic, animal model, co-expression network, and functional genomics studies relating to the high confidence ASD risk gene, *CHD8*. *CHD8*, a chromatin remodeling factor, may serve as a “master regulator” of a common ASD etiology. Individuals with a *CHD8* mutation show an ASD subtype that includes similar physical characteristics, such as macrocephaly and prolonged GI problems including recurrent constipation. Similarly, animal models of *CHD8* disruption exhibit enlarged head circumference and reduced gut motility phenotypes. Systems biology approaches suggest *CHD8* and other candidate ASD risk genes are enriched during mid-fetal development, which may represent a critical time window in ASD etiology. Transcription and CHD8 binding site profiles from cell and primary tissue models of early development indicate that *CHD8* may also positively regulate other candidate ASD risk genes through both direct and indirect means. However, continued study is needed to elucidate the mechanism of regulation as well as identify which CHD8 targets are most relevant to ASD risk. Overall, these initial studies suggest the potential for common ASD etiologies and the development of personalized treatments in the future.

## Introduction

Autism spectrum disorder (ASD) is a lifelong neurodevelopmental disorder characterized by restricted, repetitive behaviors and impaired communication and social interactions (American Psychaitric Association, 2013). ASD can have a considerable impact on quality of life as many people with ASD experience difficulties communicating, developing relationships, and managing restrictive behaviors (American Psychaitric Association, 2013). Additionally, the prevalence of ASD appears to have steadily increased over the last few decades, from < 0.5% of American school aged children in the 1970s to 2% in 2012 (Blumberg et al., 2013). The most recent estimates for the median worldwide prevalence of autism and pervasive developmental disorders is about 1 in 160 children (Elsabbagh et al., 2012) and 1 in 68 children in the US (Centers for Disease Control, 2014). The apparent rise in ASD has prompted a great deal of interest and research into identifying the underlying causes of the disorder.

Many studies have indicated a strong genetic contribution for ASD risk (Geschwind and State, 2015). Early twin and familial studies demonstrated compelling evidence for the heritability of ASD due to the high rate of concordance for ASD between monozygotic twins (0.62–0.94) as opposed to dizygotic twins (0.05–0.62) and increased relative recurrence risk for siblings of affected individuals (10.1%; Abrahams and Geschwind, 2008; Risch et al., 2014; Colvert et al., 2015). However, the overall ASD genetic architecture is complex with (1) risk being conferred by *many* independent genomic loci and contributions from common and rare variants, as well as new or *de novo* mutations, (2) factors that range in size, from single base changes to large chromosomal deletions/duplications or other rearrangements, (3) impacts that include necessary and sufficient single variants, to small (oligogenic) and large (polygenic) sets of factors, (4) the relative contributions of these factors are likely different in subpopulations, such as simplex (single sporadic presentation in an individual, with no previous family history) vs. multiplex (multiple affected individuals in a family), or other demographics yet to be elucidated. These realities have hampered traditional gene discovery methods.

Despite these challenges, recent advances in sequencing technology and novel approaches have begun to unlock the genetics of idiopathic ASD and *high confidence* risk genes are beginning to emerge that shed new light on the underlying biology of this disorder. Here, we highlight one such gene, *Chromodomain helicase binding protein 8 (CHD8)*. We review the discovery of *CHD8* as one of the most mutated genes in simplex ASD, its molecular function, associated ASD subtype, and its potential role as a master regulator of other candidate ASD risk genes. Although *CHD8* is still just one of many ASD risk genes, these data point toward at least one converging neuromolecular mechanism in ASD etiology and the potential stratification of ASD into distinct genetic/biologic subtypes. This promises to have major implications for the future as we strive to develop personalized therapies and precision treatments for individuals with ASD.

## Genomic discovery revolution

Earlier comparative genomic hybridization and genotyping array studies advanced the genomewide discovery of large regions of chromosomal deletions or duplications, termed copy number variants (CNVs), as substantial contributors to the risk of developing neurodevelopmental disorders (Rosenfeld et al., 2010). CNVs typically involve gains or losses of many genes and can show diversity in penetrance and expressivity. While these CNVs play a critical role in the architecture of neurodevelopmental disorders, with few expectations, it has been difficult to get at the underlying locus or loci that are responsible for this risk. The advent of whole-exome (the entire protein coding exons of the genome) sequencing allowed for mutation detection at single-base resolution and was initially applied to Mendelian disorders (Ng et al., 2010). A number of groups began applying these methods to small cohorts of sporadic cases of a variety of conditions, including idiopathic intellectual disability (ID), ASD, and schizophrenia, focusing on parent-child trios or families (Vissers et al., 2010; Girard et al., 2011; O'Roak et al., 2011; Xu et al., 2011). It was hypothesized that such families would likely be enriched for *de novo* mutations related to the condition, thereby allowing the identification of novel genetic events with major biologic effect in an unbiased genomwide fashion. These initial studies demonstrated the feasibility of obtaining exome data of sufficient quality across the trios (>90% jointly covered) and filtering strategies to identify the ~1 true *de novo* event expected per generation (Veltman and Brunner, 2012). Moreover, they appeared to yield a large number of *possible* candidate genes for these conditions.

For simplex or sporadic ASD, these efforts were greatly expanded in 2012 with four groups publishing the results of hundreds of families independently (Iossifov et al., 2012; Neale et al., 2012; O'Roak et al., 2012b; Sanders et al., 2012). Mutations occurred in genes with very diverse functions and in >900 ASD probands (affected individuals) only six genes exhibited recurrent likely loss-of-function (LoF) mutations, defined as nonsense (premature stop codon resulting in gene truncation), canonical splice-site (altered donor or acceptor splice sites leading to improperly spliced mRNA) or frameshifting nucleotide insertion/deletion (indel), suggesting extreme locus heterogeneity. One of the genes identified with two LoF mutations in 209 ASD affected children from the Simons Simplex Collection (SSC) by O'Roak and colleagues was *CHD8*, an ATP-dependent chromatin-remodeling factor (O'Roak et al., 2012b; Table 1). CHD8 protein was also part of a large protein-protein interaction network emerging from the LoF and most severe missense mutations. This network was ranked highly for similarity to previously identified ASD risk genes using a network walking approach. Furthermore, the locus specific mutation rate for *CHD8* suggests that it was highly unlikely to find two independent mutations at random (in the single study), providing additional evidence for *CHD8* as an ASD risk factor (O'Roak et al., 2012b). Mutation of *CHD8* had not been previously implicated with ASD, except in one concurrent study examining balanced chromosomal abnormalities in ASD and other neurodevelopmental disorders (Talkowski et al., 2012). Talkowski and colleagues mapped a balanced translocation involving 3q25.31 and 14q11.1 [46 XX, *t*_(3;14)_ (q25.31;q11.2)dn], which directly disrupted only one gene, *CHD8* at the 14q11.1 breakpoint. The proband was diagnosed with ASD, ID, and had dysmorphic facial features. Large *de novo* deletions of 14q11.2 were previously observed in three other subjects that all exhibited developmental delay (DD) and cognitive impairment and similar dysmorphic features including widely spaced eyes, short nose with broad nasal tip, and unusual helical root formation of the ear (Zahir et al., 2007). The presence or absence of ASD was not noted. The overlapping regions lost in these three cases included *CHD8* and *SUPT16H*. *CHD8* was suggested as a possible candidate gene for these abnormalities (Zahir et al., 2007).

**Table 1:** *****De Novo CHD8*** Mutations**.

**Chr 14 Position**	**Ref^a^ Allele**	**Alt^a^ Allele**	**Mutation Location (HGVS)^b^**	**Mutation^c^ Type**	**Diagnosis^d^**	**VIQ^e^**	**NVIQ^f^**	**FSIQ^g^**	**Database/Cohort^h^**	**Author, Year**
2,189,9618	G	C	p.Ser62X	Ns	ASD	75	78	74	SSC	O'Roak et al., 2012a
21,899,168	C	T	p.Arg212Gln	Ms	ASD		72		TASC	O'Roak et al., 2014
21,895,989	del(47)^†^	A	c.1593_1601+38del^†^	Ssv	ASD				SSC	Iossifov et al., 2014
21,882,516	G	T	p.Gln696Lys	Ms	ASD	88	125		TASC	O'Roak et al., 2014
21,878,133	G	GT	p.Tyr747X	Fs	ASD	25	38	32	SSC	O'Roak et al., 2012a
21,876,700	A	G	p.Leu834Pro	Ms	ASD				ASC	De Rubeis et al., 2014
21,876,489	C	T	p.Met904Ile	Ms	ASD/ID		63		TASC	O'Roak et al., 2014
21,871,373	T	C	c.3519-2A>G	Sp	ASD	37	47	43	SSC	O'Roak et al., 2012a
21,871,790	C	A	p.Glu1114X	Ns	ASD	27	41	34	APP	Bernier et al., 2014
21,871,178	G	A	p.Gln1238X	Ns	ASD	20	34	27	SSC	O'Roak et al., 2012b
21,870,652	C	T	p.Arg1242Gln	Ms	ASD				ASC	De Rubeis et al., 2014
21,870,169	G	A	p.Arg1337X	Ns	ASD	85	86	84	SSC	O'Roak et al., 2012a
21,868,219	G	A	p.Arg1580Trp	Ms	ASD	97	74		TASC	O'Roak et al., 2014
21,867,866	T	G	p.Tyr1642LeufsX25	Ssv-Fs	ASD				ASC	De Rubeis et al., 2014
21,867,866	T	G	c.4818-2A>C	Ssv	ASD		93		TASC	O'Roak et al., 2014
21,865,980	A	T	p.Ser1606ArgfsX8	Ssv-Fs	ASD				ASC	De Rubeis et al., 2014
21,865,980	A	T	c.5051+2T>A	Ssv	ASD	96	103		TASC	O'Roak et al., 2014
21,862,642	C	T	p.Gly1602ValfsX15	Ssv-Fs	ASD				ASC	De Rubeis et al., 2014
21,862,535	G	A	p.Arg1834X	Ns	ASD				TASC	O'Roak et al., 2014
21,862,159	CC	C	p.Glu1932SerfsX3	Fs	DD/ID/ASD			46	Troina	Bernier et al., 2014
21,861,643	TCTTC	T	p.Glu2103ArgfsX3	Fs	ASD	44	67	59	SSC	O'Roak et al., 2012a
21,861,376	ACT	A	p.Leu2120ProfsX13	Fs	ASD	90	93	91	SSC	O'Roak et al., 2012b
21,860,919	C	A	p.Ser2173X	Ns	ASD/SHZ					Mccarthy et al., 2014
21,861,328	T	TC	p.Glu2136ArgfsX6	Fs	ID			<40	Troina	Bernier et al., 2014
21,854,022	GGGT	G	p.His2498del	Aa	ASD	84	98	92	SSC	O'Roak et al., 2012a
			t.3;14,q25.31;q11.2	Tr	ASD/ID				AGRE	Talkowski et al., 2012

a *VCF format, hg19 coordinates*.

b *Accession number: NP_001164100.1; Ref Seq number: NM_001170629.1*.

c *Fs, Frameshift; Ns, Nonsense; Tr, Translocation; Aa, Single amino acid deletion; Sp, Splice; Mns, Missense near splice site; Ms, Missense; Ssv, Splice site variant*.

d *ASD, Autism Spectrum Disorder; ID, Intellectual Disability; SHZ, Schizophrenia*.

e *VIQ, Verbal I.Q*.

f *NVIQ, Non Verbal I.Q*.

g *FSIQ, Full Scale I.Q*.

h *SSC, Simons Simplex Collection; AGRE, Autism Research Genome Exchange; ASC, Autism Sequencing Consortium; TASC, The Autism Simplex Collection*.

† *CAAGCTCAAGTGAGTACTCCTTGCTACTGTGATGGGACGT*.

In an effort to identify additional mutations and firmly implicate *CHD8* and other strong candidates, targeted re-sequencing studies utilizing cost-effective modified molecular inversion probes (MIPs) were applied to larger cohorts of ASD and DD probands. In the first such study, the protein coding regions of 44 genes were successfully sequenced in 2446 ASD probands from the SSC (O'Roak et al., 2012a). Seven additional *de novo CHD8* mutations were identified: six LoF variants and a single amino acid deletion, including a mutation missed by exome sequencing (Table 1). This finding was highly significant as determined by a model of recurrent gene mutation, firmly implicating *de novo* LoF mutations in *CHD8* with ASD risk (*p* < 2 × 10^−9^). Including exome data available at the time, 9/2573 (0.35%) SSC probands carried a *CHD8* mutation, making this locus one of the most frequently mutated in sporadic/simplex ASD.

These resequencing studies were further extended in a broader neurodevelopmental cohort of 3730 children with ASD or DD (Bernier et al., 2014), 898 ASD confirmed probands from The Simplex Autism Collection (TASC; O'Roak et al., 2014), as well as ~2500 unaffected children from SSC or TASC. These studies identified six additional *de novo* LoF variants, four *de novo* missense variants, and one inherited LoF variant. LoF mutations were not seen in unaffected siblings or in an additional 6503 general population control (Bernier et al., 2014; O'Roak et al., 2014). In total, 16 *de novo CHD8* mutations (0.46%, Poisson 95% CI: 0.26–0.75%) were identified in the SSC and TASC cohorts, both of which required probands to meet ASD criteria on the Autism Diagnostic Interview-Revised (ADI-R) and Autism Diagnostic Observation Schedule (ADOS; Lord et al., 1994, 1999). Combined with the more broadly defined Bernier et al. cohort, the observed *de novo* rate is 0.3%. These findings further strengthen the association of *de novo* mutations in *CHD8* with ASD risk by identifying mutations in independent cohorts.

In late 2014, two large-scale exome sequence studies were also published, including ~2500 families from the SSC (Iossifov et al., 2014) and 1478 families and 1673 case-only by the Autism Sequencing Consortium (De Rubeis et al., 2014). In the SSC one *new* additional *CHD8* mutation was identified (nine previously identified including MIP and exome data). The ASC identified two *CHD8 de novo* missense mutations from the family and three splice site variants from case only exome data (through genotyping selected parents; De Rubeis et al., 2014). Risk genes were identified using a statistical approach called TADA (Transmission and *De Novo* Association), that integrates family-based (de novo and transmitted) and case-control data (He et al., 2013). In this analysis, *CHD8* was also identified as one of the top 13 ASD risk genes with a false discovery rate (FDR) of < 0.01. In summation, both exome and targeted resequening data have firmly demonstrated that *de novo CHD8* mutations play an important role in ASD risk.

## *CHD8* mutations define a subtype of ASD

*CHD8* is not only one of the most recurrently mutated genes in sporadic ASD, but also appears to give rise to a distinct ASD phenotype. The first two probands with *CHD8* mutations identified from the early sequencing efforts of O'Roak and colleagues, interestingly, had unusually large head circumferences (macrocephaly; O'Roak et al., 2012b). Similarly, the index case from Talkwoski et al. carrying the *CHD8* disrupting balanced translocation also presented with macrocephaly and had dysmorphic facial features including prominent forehead and eyes and posteriorly rotated ears (Talkowski et al., 2012). Noting this, head circumference of the the eight probands with LoF mutations in *CHD8* identified from targeted re-sequencing of the SSC (O'Roak et al., 2012a) was examined. They found that head size was significantly larger in the individuals with *CHD8* mutations (greater than two standard deviations) as opposed to those without. Additionally, about 2% of SSC probands with macrocephay had *CHD8* mutations. Macrocephaly has been recognized in other genetic etiologies of ASD, mainly loss of *PTEN* function and deletions in 16p11.2 (Butler et al., 2005; Shinawi et al., 2010).

**Figure 1:**
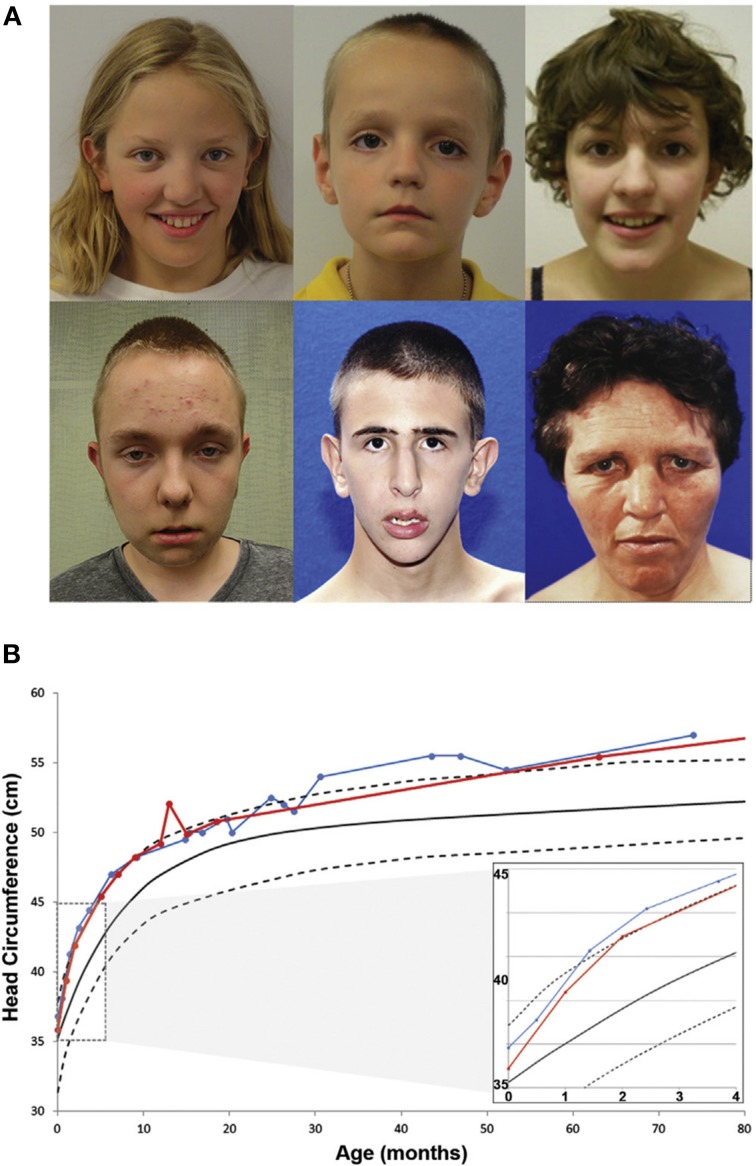
**Phenotypic characteristics of patients with *CHD8* Mutations**. **(A)** Common facial features of patients with *CHD8* mutations include macrocephaly, hypertelorism, down-slanted palpebral fissures, broad nose, pointed chin, and prominent supra-orbital ridge. **(B)** Longitudinal head circumference data for two patients (red and blue). At 2 months after birth, orbital frontal head growth is pronounced. Head growth continues to be in the 97th percentile throughout childhood. Figure originally published in Bernier et al. (2014) used with permission.

To expand the group of individuals with known *CHD8* mutations and assess the potential for a *CHD8* related subphenotype, patients identified through the exome and targeted sequencing ASD and DD cohorts were invited to participate in a comprehensive structured evaluation using a battery of standard cognitive, adaptive, and language tests (*n* = 8; Bernier et al., 2014). Including previous clinical reports on other subjects (*n* = 7), phenotypic data on 15 total patients with disruptive *CHD8* variants were included in the evaluation (13 *de novo*, one inherited, and one of unknown origin; Figure 1A and Table 2). ASD was the most common diagnosis with 13 of 15 meeting a strict diagnosis on both “gold standard approaches,” ADI-R and ADOS. Macrocephaly, defined as orbitofrontal circumference greater than two standard deviations of age and sex matched means, was exhibited in 12 of 15 patients (Table 2). Head circumference velocity data (*n* = 2) showed an initial orbital overgrowth within the first 2 months post birth and a continued trajectory of large head growth at or above the 97th percentile throughout early childhood (Figure 1B). The proportion of *CHD8* mutation carriers with macrocephaly is significantly greater than that seen in the typical ASD population (*p* = 2.1 × 10^−21^). There were also many similarities in facial features among the group including: prominent forehead, wide set eyes, broad nose with full nasal tip, and pointed chin (Figure 1A). A majority also reported gastrointestinal (GI) problems, including recurrent constipation, and sleep problems, particularly with falling asleep (Table 2). Two patients described suffering from an inability to sleep for two-three straight days. Cognitive impairment was also pervasive, but intelligence spanned a wide range with some in normal range. Moreover, there is indication that additional symptoms may manifest as the children age. Three of the three female patients (including the translocation case) experienced precocious puberty (Talkowski et al., 2012; Bernier et al., 2014).

**Table 2:** **Phenotypic Summary of Patients with CHD8 Mutations**.

**Patient characteristics**	**Number (%)**
	
ASD	13/15 (87%)
Tall stature	12/14 (86%)
Macrocephaly^*^	12/15 (80%)
GI problems^*^	12/15 (80%)
Sleep problems	10/15 (67%)
Intellectual disability	9/15 (60%)

* *Indicates significantly different from the typical ASD population*.

The implications of a *CHD8* specific phenotype suggest ASD could be stratified into different subtypes based on genetic etiology. Identification of these subtypes would provide insight into ASD presentation and pathology in genetically homogenous populations and aid in the development and evaluation of treatment approaches (Bernier et al., 2014). Other high confidence ASD risk genes that exhibit recurrent loss of function mutations, such as *ADNP* and *DYRK1A* also appear to be associated with emerging subphenotypes (Helsmoortel et al., 2014; Bronicki et al., 2015; Ji et al., 2015). *CHD8* appears to have a great deal of specificity for the ASD phenotype as almost all individuals identified from the screen met the criteria for ASD even though the cohort was not strictly defined for ASD individuals. This however may often not be the case as other strong ASD risk genes show more variable expressivity. For example, *SYNGAP1* and *CHD2* have been independently implicated in other neurological disorders such as epileptic encephalopathies (Carvill et al., 2013). Future studies examining how these similar genetic mutations span our current diagnostic and clinical boundaries may provide novel insights into the development of common neuropathologies.

## A chromatin modifier with diverse functions

*CHD8*, previously called Duplin, was first identified in a screen for novel interactors within the canonical Wnt/β-catenin pathway using a yeast two-hybrid assay with a cDNA library generated from rat brain (Sakamoto et al., 2000). *CHD8* is a member of the chromodomain-helicase-DNA binding protein family. The CHD family is characterized by a SNF-2 like ATPase and two chromo (chromatin organization modifier) domains (Marfella and Imbalzano, 2007). The first CHD gene, *CHD1*, was identified in a search for Ig promoter binding proteins (Delmas et al., 1993; Woodage et al., 1997) At least nine other members have been characterized to date with varying patterns of expression in timing and localization. The CHD family is a structurally diverse group comprised of three main subfamilies categorized by the presence of certain functional domains (Marfella and Imbalzano, 2007). CHD1 and CHD2 proteins contain DNA binding domains near the C-terminus that are thought to recognize AT rich motifs. CHD3 and CHD4 lack DNA binding domains and instead have PHD (plant homeo domain) Zinc finger like domains that may recognize methylated histones and potentially have repressive functions. CHD5-CHD9 also have DNA binding domains, along with additional functional domains located at the C-terminus. CHD8, as well as CHD7 and CHD9, contain a BRK domain, which is often seen in other SWI/SNF like proteins, and a DNA binding domain. Other SWI/SNF like complexes, such as BAF, regulate neural tube closure and neuron progenitor identity and differentiation (Ronan et al., 2013). Based on its ATP-dependent chromatin remodeling capabilities and SWI/SNF like domains, a potential role for CHD8 may lie in the regulation of neuronal progenitor cells.

Studies of CHD8 protein in the context of human cellular function show that it is involved in the regulation of transcription factor activity. CHD8 is able to activate the transcription of genes driven by U6 promoters through interactions with the transcription factor hStaf (Yuan et al., 2007). CHD8 is also required for CTCF, a major transcriptional repressor, to function as an inhibitor and insulator (Ishihara et al., 2006). CHD8 can also regulate RNA synthesis as well due to interactions with RNA polymerase II (Rodríguez-Paredes et al., 2009). CHD8 also promotes cellular proliferation in multiple human-derived cell types. Cells with *CHD8* knockdown were found to have highly reduced S-phase cell populations and increased populations of cells arresting in G1 (Rodríguez-Paredes et al., 2009). This effect was later found to be a result of CHD8 binding to S-phase-dependent promoters and recruiting E2F, a transcription factor which controls cell cycle regulation (Subtil-Rodríguez et al., 2014). CHD8's role in cell cycle regulation could be a contributing factor in the observed macrocephaly exhibited by individuals with *CHD8* LoF mutations. While macrocephaly may suggest overproliferation, in mouse models deficient in PTEN, macrocephaly appears to be attributed to increased cell body or soma size. Similarly, if cells are unable to progress through the cell cycle due to CHD8 loss, they may continue to grow in size (Kwon et al., 2001; Luikart et al., 2011). However, both CHD8 upregulation and downregulation has been observed in cancers suggesting CHD8 cell cycle regulation is likely complex and further study will be needed to determine how it impacts neurodevelopment (Lawrence et al., 2014; Subtil-Rodríguez et al., 2014).

What is also still unclear is the mechanism by which CHD8 controls transcription factor activity. Many studies have demonstrated that CHD8 has affinity for histone H3 lysine 4 (H3K4me3), a marker for active transcription, and is present at active promoters (Yuan et al., 2007; Rodríguez-Paredes et al., 2009; Sugathan et al., 2014; Cotney et al., 2015). Additionally the loss of CHD8 protein was associated with CpG hypermethylation and histone hypoacetylation near CTCF binding sites. Though CHD8 did not affect the ability of CTCF to bind DNA, potentially the CTCF-CHD8 complex may interact with methyltransferases and histone acetyltransferases to influence the state of nearby chromatin (Ishihara et al., 2006). Thus, CHD8 could promote transcription factor binding by making chromatin more accessible or acting as a scaffold between H3K4me3 labeled histones and the transcription factor.

## *CHD8* disruption in animal models mimics human phenotypes

In the *in vivo* setting, *CHD8* was first examined in the context of β-catenin signaling due to its nuclear localization and ability to repress β-catenin dependent transcription (Sakamoto et al., 2000). In zebrafish, β-catenin is necessary for axis formation. Dorsal injections of *Chd8* mRNA, causing overexpression of the protein, resulted in loss of the head. *CHD8* expression in the zebrafish is enriched in the brain and spinal cord at 1 day post fertilization, and becomes progressively restricted to head and gut after 3–4 days post fertilization. Expression appears strongest in the mid-gastrula stage suggesting an important role for *CHD8* in embryonic development regulating Wnt/β-catenin activity (Sakamoto et al., 2000).

The association of macrocephaly with a reduction in *Chd8* expression is also observed in zebrafish. An increase in the interorbital distance, a surrogate measurement for head circumference, is seen in fish treated with *Chd8* targeting morpholinos (Bernier et al., 2014; Sugathan et al., 2014). The distance increases in a morpholino dose dependent fashion. The reduction in *CHD8* also coincides with increases in chordin, required in forebrain development, otx2, early marker of midbrain/forebrain neural progenitor cells, and HuC/D, marker for newborn neurons, indicating elevated proliferation, particularly in the mid and forebrain (Bernier et al., 2014). The zebrafish studies also suggest a role for *CHD8* in GI development. Many of the human patients with *CHD8* mutations suffer from severe GI issues. Morpholino treated zebrafish have decreased gut motility as evidenced by delayed progression of fluorescent microspheres (Bernier et al., 2014). There is also an overall reduction in enteric neurons in the hindgut.

Similarly in mice, *Chd8* expression is at its highest embryonically at E8.5–E12.5 but reduced by E16.5 and low in newborns. Therefore, function is likely restricted to early-middle stage embryonic development (Nishiyama et al., 2004). Expression of *Chd8* is most prominent in the brain, face, branchial arches, limb buds, and tail. Complete knockout of *Chd8* is embryonic lethal. Embryos are resorbed by E9.5 but still recoverable by E8.5. Growth begins to delay as soon as E5.5 and is completely arrested at E6.5 with pronounced apoptosis occurring at E7.5. Heterozygotes though did not have gross abnormalities and were fertile. Interestingly, Wnt/β-catenin targets are not increased suggesting that aberrant signaling is likely not the cause for lethality. Rather, Chd8 protein regulates p53 activity by recruiting histone H1, forming a p53-Chd8-H1 complex that represses p53 target gene expression (Nishiyama et al., 2009). Unrestrained p53 activity appears to be the cause for the massive apoptosis seen in embryos. Deletion of p53 in *Chd8*^−∕−^ mice delayed arrest to E10.5 at which time embryos died from a heart defect. *Chd8*^−∕−^ p53^+∕−^ mice did not recover as well. Taken together, these studies demonstrate a conserved role for *CHD8* as a regulator of early formative developmental pathways.

## A *CHD8*-ASD developmental window

With the great functional diversity observed in the *de novo*, rare variant, and other candidate ASD risk genes, it seems difficult to reconcile their role in an overarching mechanism for ASD biology. However, a number of biologic network approaches suggest that there may be a converging biology at particular developmental windows and brain regions, which includes *CHD8* as a central player. These approaches are leveraging new spatial and temporal genomics data from developing human and non-human primates, such as the BrainSpan Atlas, a freely available resource that includes gene expression (microarray and RNA-seq) and *in-situ* hybridization data sets (Shen et al., 2012). As in other organisms, *CHD8* expression seems to be most significant early in human development. While *CHD8* is widely expressed in the adult brain, expression is highest at 9–16 post conception weeks (PCW) in both progenitor and post mitotic neocortical layers and then gradually declines (Bernier et al., 2014).

To discover time points of development and brain regions for which candidate and high-confidence ASD risk genes, such as *CHD8*, may converge, two recent concurrent studies made use of BrainSpan's gene expression data to develop co-expression networks or modules representing genes that share similar expression patterns during development (Parikshak et al., 2013; Willsey et al., 2013). Each of these networks or modules corresponded to different brain regions along progressing stages of development. Remarkably, while the groups took different approaches to develop these networks and assess the potential for enrichment of candidate ASD risk genes they arrived at similar results implicating the mid-fetal time periods as a possible critical window for ASD etiology.

Parikshak and colleagues used BrainSpan RNA-seq data from human neocortex 8 PCW to 12 months after birth to construct co-expression modules by Weighted Gene Coexpression Network Analysis (WGCNA), an unbiased, genomewide method for constructing networks based on pairwise correlations of gene expression (Langfelder and Horvath, 2008; Parikshak et al., 2013).

They identified 17 modules and mapped sets of candidate ASD risk genes onto proteins within the modules. These gene sets included: “asdM12” which was derived from a WGCNA expression module from ASD patient postmortem cerebral cortex and “SFARI ASD” which was a subset of 155 genes from the larger AutDB gene list filtered by gene evidence score (Voineagu et al., 2011; see Table 3 for detailed gene list descriptions). *De novo* variants identified from 2012 whole-exome sequencing studies performed by O'Roak et al., Sanders et al., and Neale et al. were also mapped onto networks. They then assessed enrichment for candidate ASD risk gene within specific co-expression modules, cortical layers and cell types. The *de novo* mutation candidate ASD risk genes were more concentrated in modules designated M2 (*p* = 0.006) and M3 (*p* = 0.0011). M2 peaks at 12–22 PCW and M3 is highly upregulated until PCW 12. The overall set of genes in these modules, including non-ASD risk genes, were highly enriched for chromatin modifiers, DNA binding proteins, and transcriptional regulators (*p* < 1 × 10^−4^). Modules M2 and M3 seemed specific to the superficial cortical layers L2–L4 and cells expressing glutamatergic neurons.

**Table 3:** **Referenced Candidate ASD Risk Gene Lists**.

**Gene list name**	**Compilation method**	**Type**	**Number of genes**	**Author, Year**
AutDB	“PubMed” database search for “gene” + “autism” or “autistic” in the titles and abstracts. Genes are divided into genetic subcategories and assigned evidence scores. Licensed to SFARI as SFARI Gene by MindSPec.	Broad	667	Basu et al., 2009
asdM12	Set of genes within a co-expression module (“M12”) highly correlated with ASD status. Module was constructed using WGCNA on gene expression from ASD (19) and control (17) postmortem cortex tissue. Samples from Autism Tissue Bank and Harvard Brain Bank.	Co-expression	88	Voineagu et al., 2011
SFARI ASD	Subset of genes from AutDB filtered for gene category syndromic (S) (associated with syndromes in which a significant percentage of individuals develop autistic symptoms) and evidence score 1–4 (high confidence—minimal evidence).	Broad	155	Parikshak et al., 2013
Willsey set	Genes identified with *de novo* LoF mutations from whole-exome sequencing studies (Iossifov et al., 2012; Kong et al., 2012; Neale et al., 2012; O'Roak et al., 2012a) and an additional 56 quartets from the SSC. Genes are grouped as, high confident (hcASD) genes (2 or more *de novo* LoF mutations), or probable (pASD) genes (one *de novo* LoF mutation). The genes in the pASD group were estimated to have a >50% chance of being “true ASD” genes based on TADA analysis.	Simplex dnLoF	131	Willsey et al., 2013
Liu set	Genes identified by the DAWN algorithm as being implicated in ASD risk using data from the PFC-MSC co-expression module developed in Willsey et al. and from whole-exome sequencing studies in family trios (Iossifov et al., 2012; Kong et al., 2012; Neale et al., 2012; O'Roak et al., 2012a; Sanders et al., 2012), quartets (Willsey et al., 2013) and case-controls (ARRA Autism Sequencing Consortium, De Rubeis et al., 2014)	dnLoF Rare transmitted variants Co-expression	127	Liu et al., 2014

In contrast, the “SFARI ASD” and “asdM12” gene sets were enriched in modules M13 (SFARI ASD, *p* = 0.059, asdM12, *p* = 3 × 10^−15^), M16 (SFARI ASD, *p* = 0.0024, asdM12, *p* = 3.5 × 10^−15^), and M17 (SFARI ASD, *p* = 0.033, asdM12, *p* = 1.0 × 10^−7^). The overall set of genes in these modules had a concentration of synaptic proteins (*p* < 1 × 10^−4^) and peaks late in fetal development, starting at 16 PCW and into birth. An enrichment for fragile-X mental retardation protein (FMRP) targets was seen in the M2 module, suggesting a common mechanism between ASD and fragile-X syndrome, in line with the enrichment of FMRP targets first observed by Iossifov et al. (2012). On the other hand, 401 genes implicated in monogenic forms of ID compiled from four publications, showed little enrichment for any one module suggesting the mid-fetal time period may be specific to ASD.

Willsey and colleagues took an alternative approach by focusing on nine high-confidence ASD risk genes, defined as having two or more LoF mutations, and determining their co-expression networks (Willsey et al., 2013; Table 3). These nine genes were used as seeds to generate networks composed of the top positively correlated genes. Gene expression data was also derived from BrainSpan, but based on the Affymetrix GeneChip Human Exon 1.0 ST Array rather than RNA-seq. In addition, all brain regions were considered and 15 periods of development, 5.7 PCW–82 years were examined. Developmental periods were condensed into three time windows resulting in 52 networks based on time and brain region with transcriptional similarity as determined by hierarchical clustering. To see if additional candidate ASD risk genes were enriched in these networks, a list of 122 candidate ASD genes (termed “Willsey set”) with at least one LoF mutation identified from whole-exome sequencing studies was generated and mapped onto the networks (Table 3). Permutation testing of enriched networks was used to correct for gene size and GC content. They also looked for enrichment of this gene set within specific cortical layers and cell types. Immunostaining and *in-situ* hybridization was performed on frontal cortex sections to determine protein expression for five of the high-confidence ASD genes. Similar to Parikshak et al., they found enrichment in networks exhibiting peak expression between 10–19 (*p* = 0.003) and 13–24 (*p* = 0.05) PCW (mid-fetal time period). These networks were specific to the prefrontal and primary motor-somatosensory cortex, but in contrast to Parikshak et al., the networks were specific to the deep layers, L5-6. Immunostaining revealed that *CHD8* and other ASD genes like *SCN2A, DYRK1A*, and *TBR1* were highly expressed in cortical projection neurons, particularly glutamatergic cell types.

In summary, both groups found a convergence of candidate ASD genes with *de novo* mutations within the mid-fetal stage of development, 10–24 PCW. The resulting networks appeared specific for glutamaterigic cortical projection neurons, but differed in their implication of specific cortical layers. The cortical projection neurons form synaptic connections early in development and may then be highly sensitive to changes in gene regulation (Willsey et al., 2013). Therefore, *de novo* mutations in genes like *CHD8* may largely impact the midfetal stage of development particularly prefrontal and motorsensory cortex development.

## *CHD8* disruption alters many targets including other candidate ASD risk genes

**Figure 2:**
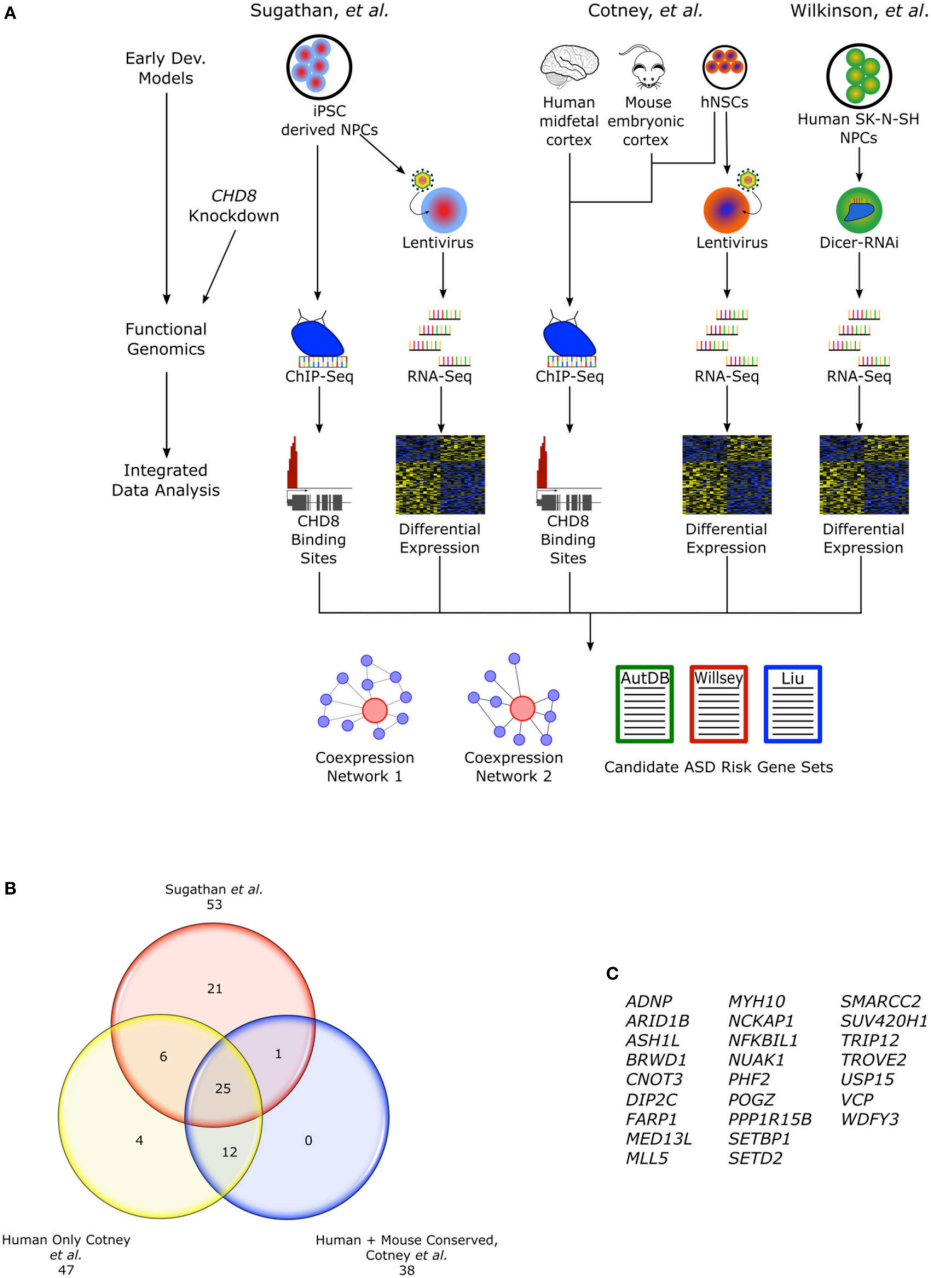
***CHD8* functional genomics studies**. **(A)** Flow chart describing functional genomics studies, Sugathan et al., Cotney et al., and Wilkinson et al. including *CHD8* knockdown in cellular models of early neural development and ChIP-seq. Cotney et al. also incorporated primary fetal human cortical and embryonic mouse brain tissue into the ChIP-seq analysis. Integrating the gene expression and CHD8 binding profiles, networks of CHD8 regulated genes were constructed and analyzed for enrichment of candidate ASD risk genes. **(B)** CHD8 targeted candidate ASD risk genes found in the “Willsey set” observed between studies. Genes found in Cotney et al. specifically have CHD8 bound promoters. Human sites in Cotney et al. are shared between hNSCs and brain tissue. Though not included as part of the “Willsey set” in Cotney et al., *POGZ* is included here. *POGZ* was noted as having a CHD8 bound promoter and it is one of the high confidence “Willsey set” genes. **(C)** List of the shared candidate ASD risk genes from the “Willsey Set” bound by CHD8 between both studies and conserved between human and mouse.

The heterozygous LoF *CHD8* mutations seen in ASD likely result in insufficient levels of CHD8 protein and the disregulation of CHD8 targets. The regulatory landscape and widespread reach of *CHD8* regulation has been examined in three recent studies using genomic approaches and a number of different neural cellular models that attempt to mimic early development (Figure 2A). Sugathan and colleagues performed knockdown of *CHD8* in induced pluripotent stem cell (iPSC) derived neuron progenitor cells (NPCs) using lentiviral delivery of six independent shRNA achieving a range of 38–69% reduction in *CHD8* mRNA expression (Sugathan et al., 2014). They then assessed changes in gene expression using RNA-seq in these knockdown lines and also performed chromatin immunoprecipitation-sequencing (ChIP-seq) in control NPCs to identify CHD8 binding sites. By contrast, Cotney and colleagues performed ChIP-seq to locate CHD8 binding sites (under non-perturbed conditions) in three different systems: human midfetal cortical tissue 16–19 PCW, H9 derived human neural stem cells (hNSCs), and embryonic mouse cortex (Cotney et al., 2015). Using primary tissue allowed for representation of mid-fetal stages critical in CHD8-ASD development and to evaluate potential differences between CHD8 binding sites identified in *in vivo* tissue and *in vitro* cells. They also performed *CHD8* knockdown in hNSCs using two lentiviral shRNA, followed with RNA-seq, to assess changes in gene expression. Finally in the third study, Wilkinson and colleagues performed knockdown of *CHD8*, achieving about a 50% reduction, using siRNA in human SK-N-SH neural progenitor cells followed by RNA-seq (Wilkinson et al., 2015). These studies reveal a complex role for *CHD8* in ASD development as it appears to regulate expression of additional candidate ASD risk genes by both indirect and direct means.

Sugathan and colleagues found that knockdown of *CHD8* lead to differential expression of some 1756 genes (at nominal *P* < 0.05; 369 genes at *q* < 0.05 Benhamini-Hochberg), most of which were upregulated (*n* = 1140, *p* < 0.05; 286, *q* < 0.05; Sugathan et al., 2014). The set of down regulated genes (*n* = 616) were enriched for roles in neuronal development. Intriguingly, this same set of downregulated genes was also enriched for candidate ASD risk genes from a large set of 628 genes from AutDB (*p* = 3.25 × 10^−8^; Table 3). Though no enrichment for any candidate ASD risk gene sets were observed in the set of up-regulated genes, cancer associated genes, defined by The Cancer Genome Atlas, were enriched in this group. Loss of *CHD8* function has been observed in a broad range of tumor types and other chromatin regulators have been implicated in cancer development (Lawrence et al., 2014). This suggests *CHD8* may have other important roles extending past development and may be a point of commonality between some cancers and ASD.

In the ChIP-seq data, 7324 reproducible binding sites were identified (Benjamini-Hochberg *q* < 0.05). CHD8 preferentially bound to regions that in ES cell-derived neuroprogenitor lines from the Roadmap Epigenomics are marked with histone H3 trimethyl Lysine4 (H3K4me3), an indicator of active TSSs (Bernstein et al., 2010). These TSSs were strongly enriched in the CHD8 set, 83%, relative to the genome background, 1%. A small number of binding sites, 4%, overlap enhancer marked sites (H3K4me1), representing a two-fold enrichment. Sequence motifs at CHD8 binding sites were enriched for CTCF and YY1 transcription factor motifs. Genes with CHD8 sites were enriched for p53, hedgehog, and cell cycle pathways.

Combining the differentially expressed (DE) set of genes with CHD8 binding data, Sugathan and colleagues further explored a number of enrichments using functional classifications, candidate ASD risk gene sets, and other disease gene sets. Overall, only 9.2% of genes with CHD8 binding sites were DE in the nominal P gene set (29.7% of all the DE genes). Candidate ASD risk genes from AutDB were enriched in the set of nominally downregulated genes, but not targeted by CHD8 (*p* = 1.09 × 10^−9^). This set of candidate ASD risk genes was enriched for pathways involved in neurodevelopment such as axon guidance and neurotransmitter regulation. Enrichment of the more restricted set of AutDB genes from Parikshak et al., “SFARI ASD” was also observed in the downregulated, non-CHD8 bound genes (*p* = 2.26 × 10^−2^). However, genes from Willsey et al., “Willsey set” were enriched as CHD8 bound genes (*p* = 4.34 × 10^−3^), but were not associated with any changes in gene expression. CHD8 bound genes in the “Willsey set” were enriched for chromatin and transcription. Though these data sets are not independent, they do have different gene representations, with the “Willsey set” more biased toward rare *de novo* variants. CHD8 binding sites with or without differential expression were explored in the modules described in Parikshak et al. The downregulated genes with no CHD8 binding site were enriched in modules M13, M16, and M17 which correspond to mid-late fetal develop whereas CHD8 bound genes were enriched in modules M2 and M3 corresponding to early-midfetal development. CHD8 bound genes were also highly enriched in TGCA cancer associated genes and also showed no specific enrichment in either up or downregulated genes. No other disease gene sets from the 184 lists for complex disease and traits available from the National Human Genome Research Institute were as significantly enriched as the ASD or cancer gene sets for CHD8 targets or genes affected by *CHD8* knockdown. These data suggest both direct and indirect roles of CHD8 in gene regulation, particularly in the development of ASD and cancer.

Cotney et al. found 9414 reproducible CHD8 binding sites in hNSCs and 4428 in human midfetal brain. Between these two human sets, 2777 sites were overlapping. Chromatin state of hNSC CHD8 binding sites was determined from data generated on hNSCs from their own lab and the ENCODE/Roadmap Epigenomics (Bernstein et al., 2010). Similarly to Sugathan and colleagues, they found that CHD8 had strong affinity for TSSs labeled with H3K4me3 or H3K27ac and 99% of promoters bound by CHD8, 8056, were active (Cotney et al., 2015). Additionally, they observed that a small fraction of CHD8 binding sites, 1028, were indicative of enhancer functions. Moreover, CHD8 binding negatively correlated with H3K27me3, indicative of repression. Enriched sequence motifs for transcription factors included CTCF, E2F, YY1, and Sp/Kruppel-like family.

Genes that had CHD8 binding sites identified in both midfetal brain and hNSCs were highly enriched for candidate ASD risk genes from two gene sets as defined by and Liu et al., “Liu set” (*p* < 0.0001) and the “Willsey set” (*p* < 0.0001; Willsey et al., 2013; Liu et al., 2014), while genes with binding sites only in hNSCs were not enriched. Candidate ASD risk genes with CHD8 binding sites were enriched for chromatin regulation and modification. CHD8 targets supported by both human datasets were largely evolutionarily conserved. Among mouse (E17.5 cortex), human midfetal brain, and hNSC, 1910 CHD8 binding sites were shared and also enriched for candidate ASD risk genes from both aforementioned gene sets (*p* < 0.0001), further suggesting a conserved mechanism for CHD8 regulation in development. Of the candidate ASD risk genes from the “Willsey set” found to contain CHD8 binding sites, 25 were shared between Cotney et al. and Sugathan et al. and were also conserved in mouse (Figures 2B,C). Enrichment of CHD8 bound proteins was also observed in networks reconstructed from the Willsey et al. 10–19 and 13–24 PCW networks using genes with active promoters (Willsey et al., 2013).

In their knockdown of *CHD8* in hNSC, genes with conserved CHD8 binding sites had the greatest fraction of DE genes (56%) as compared to genes with binding sites only found in hNSC (46%) or hNSC and human brain (54%). Cell cycle, Hippo, and p53 pathways were among the pathways most affected. Candidate ASD risk genes actually exhibited the most significant dysregulation compared to any other set of CHD8 targeted genes (~60% for both gene sets). Additionally, those candidate ASD risk genes most DE, tended to be downregulated. While Sugathan et al. did find that candidate ASD risk genes from the Willsey set were enriched as CHD8 targets, they were not significantly DE in either direction. In contrast, Cotney et al.'s data suggest that CHD8 directly *activates* this same set of candidate ASD risk genes.

In line with the other studies, Wilkinson et al. found *CHD8* reduction had a global impact on gene expression. Interestingly, the top DE genes in this study were noncoding RNAs, the majority of which were upregulated. Noncoding RNAs have only recently been appreciated as having a potential role in neurodevelopment and may be yet another layer in transcriptional control. Enrichment of candidate ASD risk genes Parikshak et al. “SFARI ASD” was only seen in the set of downregulated genes, similar to Cotney et al. as were processes involved in regulation of neuron projection, differentiation and neurogenesis (Parikshak et al., 2013; Wilkinson et al., 2015).

**Figure 3:**
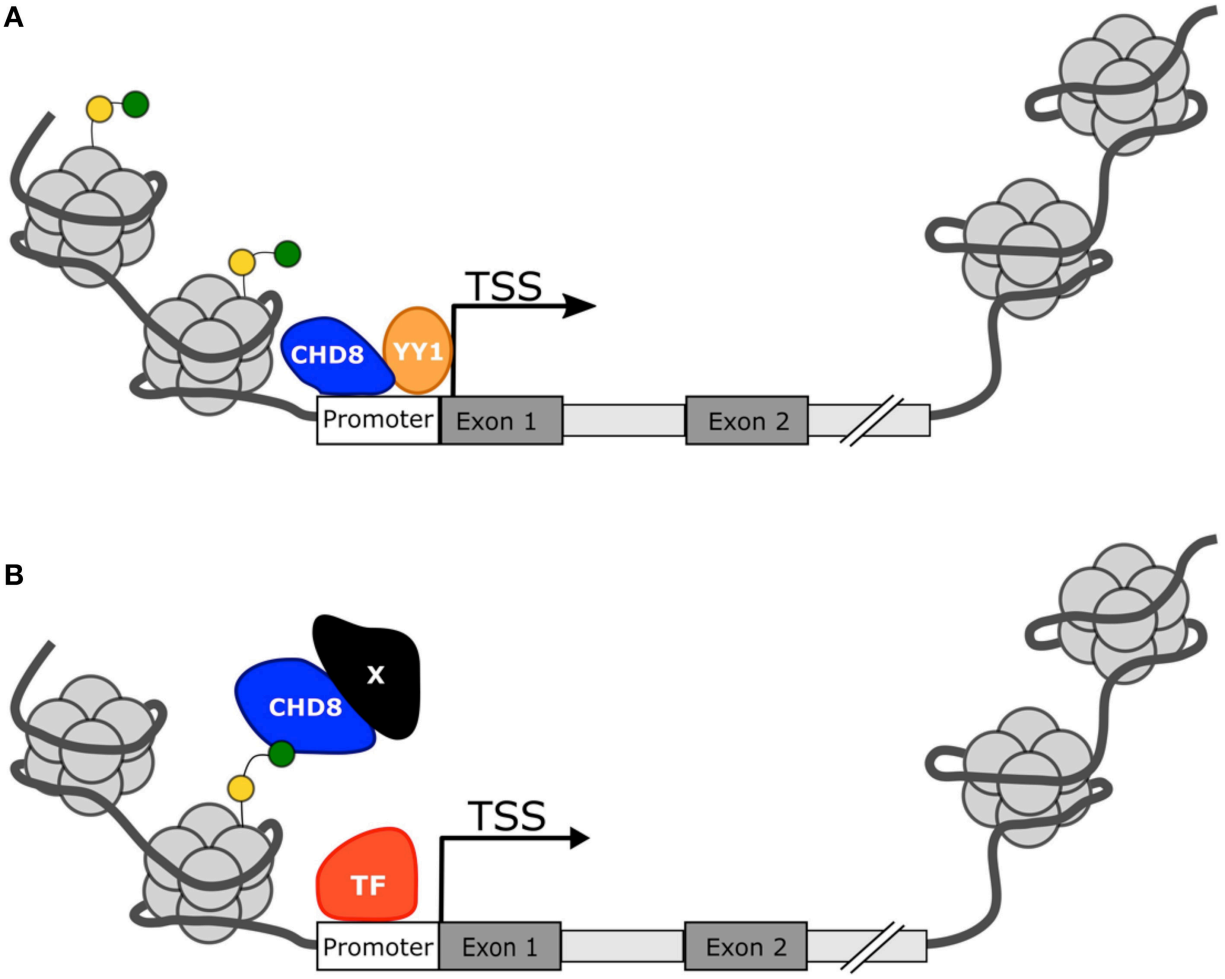
**Proposed mechanisms for CHD8 transcriptional activation**. **(A)** CHD8 is most commonly found near active transcription start sites with histone modifications H3K4me3 (green circle) or H3K27ac (yellow circle). CHD8 may directly activate genes by directly binding near the transcriptional start site and promote transcription factor activity or recruitment. **(B)** CHD8 may indirectly activate genes through interactions between modified histone sites and other co-regulators to make chromatin more assessable.

In summation, *CHD8* appears to act as a master regulator in the foundational pathways of the developing brain, particularly those that may also be implicated in ASD development. Yet the mechanism for regulation remains unclear. Many of the candidate ASD risk genes are direct targets of CHD8 protein and are also involved in chromatin regulation (Figure 3A). Cotney et al. and Wilkinson et al. indicate that CHD8 directly activates expression of these genes but Sugathan et al. suggest that the presence or absence of CHD8 may not influence expression, despite the genes having a binding site. Other candidate ASD risk genes lack a CHD8 binding site, but still appear to be affected by CHD8 expression indicating an indirect mechanism of regulation. How CHD8 may be able to influence gene activation or transcription factor activity without directly binding to DNA is not clear, but may involve binding to other co-regulators or chromatin marks (Figure 3B). Continued study will be needed to fully elucidate the mechanism for CHD8 regulation in ASD.

## Conclusions and future directions

From exome sequencing studies, a number of high-confident ASD risk genes have been identified with *CHD8* emerging as particularly strong. Both human case studies and animal models suggest a common phenotype for *CHD8* mutation including a significant association with ASD diagnosis and behavior, macrocephaly, and dysfunction in the enteric nervous system. Though ASD is highly heterogeneous, *CHD8* systems biology and functional genomics studies give some hint as to the complex nature of the disorder. The CHD8 protein and those of many of the other candidate genes serve as master regulators, influencing the expression of a large network of genes and pathways that control neuron formation, proliferation, and differentiation. The timing of proper neural development is a highly regulated phenomenon, requiring a concerted effort by many different genes. While it's clear that CHD8 controls expression of genes involved in these developmental processes, the manner in which CHD8 regulates them is still unclear. Initial studies suggest that CHD8 may act as a direct repressor by modifying chromatin to make it less accessible. However, the loss of function studies point toward a role for CHD8 as an activator in the emerging ASD gene network. One of the consistent findings from these and previous studies is that CHD8 is associated with actively transcribed genes and has affinity for H3K4me3 labeled promoters suggesting an influential role in global gene expression. CHD8 may function to make promoters more accessible through chromatin remodeling, promote transcription factor binding, or indirectly enhance expression through as of yet, unknown mechanisms that may involve interactions with H3K4me3 itself. The mechanism of CHD8 regulation is likely to be gene dependent as well.

It's also still uncertain which CHD8 targets may be relevant for ASD. Though the studies indicate a number of candidate ASD risk genes contain CHD8 binding sites, further functional studies will be required to demonstrate if these genes interact in a physiologic setting. Additionally, although enrichment for candidate ASD risk genes may be seen in specific neuron types, it is still unclear how the alterations in these neurons ultimately relate to behavioral phenotypes. It will be important to determine how *CHD8* affects neural circuitry and connect these affects to autistic associated behaviors. Lastly, development of co-expression modules have proved useful in identifying relevant ASD developmental time periods and interactions between candidate ASD risk genes, including *CHD8*. However, developing methods that integrate multiple types of data such as co-expression and protein-protein interactions may improve identification of ASD and other neurodevelopmental disorder specific networks, as proposed recently (Hormozdiari et al., 2015). Through the combination of rapidly advancing genetics and the development of relevant neural models, ASD biology is slowly beginning to resolve, and providing the potential for individualized approaches to therapy.

## Author contributions

RAB: writing, editing, and figure conception, MBP: writing and figure design, BJO: writing, editing, and figure conception.

### Conflict of interest statement

The authors declare that the research was conducted in the absence of any commercial or financial relationships that could be construed as a potential conflict of interest. Brian J. O'Roak is an inventor on patent PCT/US2009/30620: mutations in contactin associated protein 2 are associated with increased risk for idiopathic autism.
